# Analyzing annual changes in network structures of a social media application-based information-sharing system in a Japanese community

**DOI:** 10.1186/s12913-022-08478-1

**Published:** 2022-08-31

**Authors:** Junji Haruta, Sho Tsugawa, Kazunari Ogura

**Affiliations:** 1 Medical Education Center, School of Medicine, Keio University, 35 Shinanomachi, Shinjukuku, Tokyo, 160-8582 Japan; 2 Division of Information Engineering, Faculty of Engineering, Information and Systems, University of Tsukuba, Tsukuba, Japan; 3 Hachinohe Family Clinic, Hachinohe, Japan

**Keywords:** Community health services, Annual survey, Social network analysis, Interprofessional collaboration, Japan

## Abstract

**Background:**

Understanding the evolution of social network services (SNSs) can provide insights into the functions of interprofessional information-sharing systems. Using social network analysis, we aimed to analyze annual changes in the network structure of SNS-based information sharing among healthcare professionals over a 3-year period in Japan.

**Methods:**

We analyzed data on SNS-based information sharing networks with online message boards for healthcare professionals for 2018, 2019, and 2020 in a Japanese community.

These networks were created for each patient so that healthcare professionals could post and view messages on the web platform. In the social network analysis (SNA), healthcare professionals registered with a patient group were represented as nodes, and message posting and viewing relationships were represented as links. We investigated the structural characteristics of the networks using several measures for SNA, including reciprocity, assortativity and betweenness centrality, which reflect interrelational links, the prevalence of similar nodes with neighbors, and the mediating roles of other nodes, respectively. Next, to compare year-to-year trends in networks of patients overall, and between receiving nursing care levels 1–3 (lighter care requirement) and levels 4–5 (heavier care requirement), we described the annual structural differences and analyzed each measure for SNA using the Steel–Dwass test.

**Results:**

Among 844, 940, and 1063 groups in each year, groups for analysis in care levels 1–3/4–5 were identified as 106/135, 79/89, and 57/57, respectively. The overall annual assessment showed a trend toward increased diameter and decreased density, but the differences were not significant. For those requiring care levels 1–3, assortativity decreased significantly, while for those requiring care levels 4–5, reciprocity decreased and betweenness centrality increased significantly. No significant differences were found in the other items.

**Discussion:**

This study revealed that the network of patients with a lighter care requirement had more connections consisting of nodes with different links, whereas the network of patients with a heavier care requirement had more fixed intermediary roles and weaker interrelationships among healthcare professionals. Clarifying interprofessional collaborative mechanisms underlying development patterns among healthcare professionals can contribute to future clinical quality improvement.

## Introduction

With the rapid aging of the population across the world, complex health problems are arising from challenges associated with multimorbidity, aging and inactivity [1]. Japan has the world's highest proportion of elderly people, at 29.1% of the total population in 2018 [2]. The Japanese government has proposed the establishment by 2025 of a "community-based integrated care system," consisting of a medical insurance system and a long-term care insurance system, with the aim of ensuring provision of comprehensive healthcare, long-term care, preventive healthcare, housing, and lifestyle support. In this system, care services are provided in the local community, based on patient requirements. Each patient's requirements are determined by the extent of care services needed. In the current Japanese long-term care insurance system, care services are categorized into seven levels based on the condition of each patient or user. Level 5 represents the highest level of long-term care, level 1 represents the lowest, with levels 1 and 2 including plans to forestall long-term care [3]. To achieve this, healthcare professionals need to fully understand the physical and mental characteristics of the elderly, and collaborate with other professionals in a comprehensive community-based care system [4]. An interprofessional information-sharing network to solve these complex problems is needed, not only in Japan but also elsewhere in Asia and other developed countries further afield.

### Social networking services for healthcare professionals

Systems for sharing patient information are making increasing use of social networking services (SNSs), as in the example of an online message board focusing on coronary heart disease [5]. SNS-based information sharing networks can serve as an effective training tool for interprofessional collaboration [6]. SNSs have enabled the sharing of knowledge among professionals through web-based networking and the sharing of personal information with few constraints of time, space, or geography [7]. Because of expected further growth in the use of SNS-based information sharing networks as a tool for healthcare professionals in community-based integrated systems, there is a need to clarify the mechanisms and annual changes in information sharing. However, little is known about how information sharing networks develop in such SNSs for healthcare professionals [8].

### Review of social network analysis (SNA)

For analyzing network development and dynamics, social network analysis (SNA) is a useful tool [9]. SNA is a distinctive set of procedures for mapping, measuring, and analyzing social relationships among people, teams, and organizations [10]. It provides a visual representation of nodes (individuals, groups, organizations, etc.), and facilitates exploration of patterns and types of relationships among nodes. Additionally, by analyzing relationships (links) between nodes, the roles and influences of particular nodes in the network can be examined. Such SNAs can provide a theoretical approach for exploring interactions of nodes in the network based on social interaction frameworks [11] SNA has been used in a variety of ways in systematic reviews in healthcare settings [12–14]. However, in some studies SNA has been used as a descriptive tool rather than an evaluative one, and few annual analyses of information-sharing networks of healthcare professionals exist. One such study used a questionnaire design that was influenced by recall bias and other factors [15]. Revealing the development of SNS-based information sharing networks for healthcare professionals over time can help identify strengths and gaps in the network and improve the effectiveness of interprofessional collaboration [13].

Here, therefore, we aimed to reveal the annual changes in the network structures of an SNS-based information sharing system among healthcare professionals in a Japanese community over a 3-year period.

## Methods

### Design

We conducted repeated cross-sectional surveys [16]. Repeated cross-sectional data are created when a survey is administered to a new sample at successive time points. For annual surveys, this means that participants in one year may include people other than those who participated in a previous year. In the data analysis, we used SNA to analyze information sharing patterns of SNS-based networks in one community. SNA is based on the principles of graph theory [17]. It examines the existence of nodes (e.g., individual medical professionals) and internodal links, focusing on the relationships between nodes and link structures (e.g., information sharing networks). In this study, the information sharing network for each patient was constructed using an SNS-based platform that allowed different healthcare professionals to post and view messages. Based on data from 2018, we published the results of a study of the network structure and some measures of SNA, including centrality of nodes’ characteristics [18]. Those data were included in the present study for comparative purposes.

### Setting

For purposive sampling, a community within City X was selected because the healthcare professionals in the city used SNS-based platforms to share information about patients. City X is a core city in the local community, with a population of 220,000 and seven general hospitals in 2018. Since the population distribution of City X in 2018–2020 was close to the middle of Japan, we considered it to be a representative setting.

### SNS-based information sharing tools

An SNS-based information sharing tool among healthcare professionals in City X was used in this study. The SNS was created by a private company for use by healthcare professionals in a variety of roles including doctors, nurses, care workers, care managers, physical therapists, and registered dietitians. For example, home care services in the patient's home may be provided by home care staff, visiting nurses, pharmacists, doctors, and physical or occupational therapists. Home care workers, who are not state-certified, visit the patient's home to help with meals, shopping, etc. as assistance with Activity daily lives (ADLs) and Instrumental activity daily lives (IADLs). Care workers are mainly responsible for physical care such as toileting and bathing patients, providing assistance with patients’ ADLs and IADLs in home or external facilities. Clinic clerks work with other members of a small team, handling receptionist appointment requests, prescription requests, and inquiries. Medical consultants share patient information among facilities, e.g., when patients have symptoms like fever or rash, care workers consult nurses at first, and nurses report to doctors with comments and pictures via SNS to provide proper treatments or seek advice about care plans. This SNS-based platform allows multiple registered healthcare professionals to share patient-related information. The platform is also used by the staff of several medical and nursing facilities when making social welfare appointments for transportation on patients’ admission and discharge. Although patients and healthcare professionals gave written consent for their data to be used in research when they registered on the SNS, they were also guaranteed the right to refuse to participate in this study by opting out at the beginning of the study.

### Data collection

Data were obtained from patients and healthcare professionals who agreed to participate anonymously. Patients who used the online message board and their healthcare professionals linked to the board were registered as nodes. A network was created for each patient. Healthcare professionals registered in this network were allowed to post messages and mark them for viewing on the online message board of their patients. This study involved annual surveys of patient demographics and healthcare professionals. We collected the log data for message postings from users about each patient and the user's viewing marks, and applied SNA measures such as index of network structure to organize and analyze information over the period from January to December of each year 2018, 2019, and 2020. The data included gender, age, and care levels of patients registered on the message board. It included posting and viewing of messages marked as "viewed" by the posting users, who were healthcare professionals participating in the message board.

### Constructing networks

**Fig. 1:**
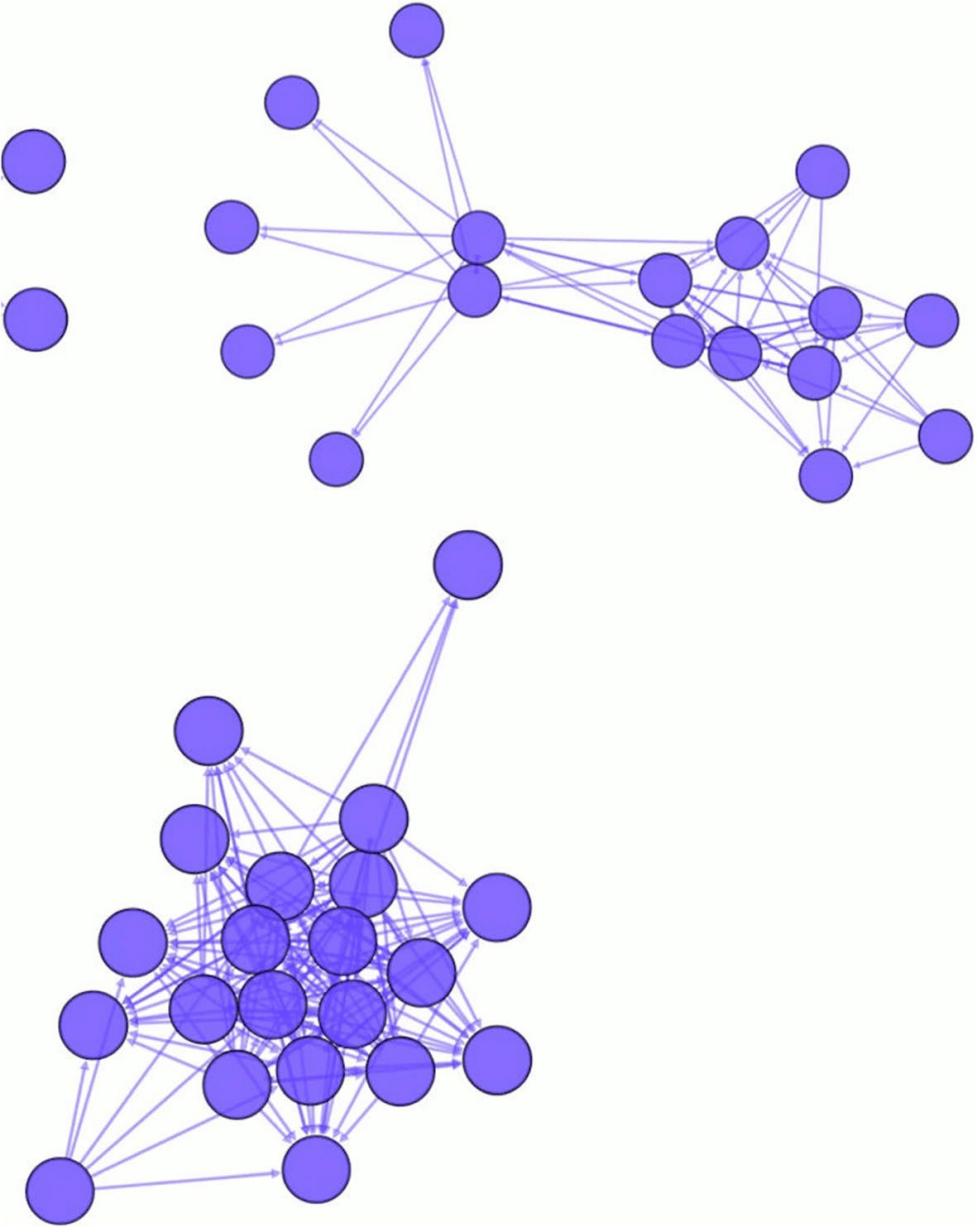
The examples of networks. An isolated node indicates that it belongs to the network but is neither input nor output.

Each group was networked from the log data of message postings from users about each patient and the user's viewing marks. Individual healthcare professionals (excluding patients) registered in a patient group were considered as nodes. Message posting/viewing relationships were considered as links. More specifically, for each patient group, an unweighted directed graph *G* = *(V, E)* was created. Node *u* represents a user (i.e., an individual medical professional)*,* and a directed link (*u, v)* represents a user *v* marked as "viewed" in a message posted by user *u.* Thus, a link was considered created when a user made a node-to-node communication through a particular thread. Because very small networks were not useful for SNA, only those with more than 10 nodes were selected for analysis, based on previous studies [19, 20]. Some of the patterns of social networks in this study may be visualized as shown in Fig. 1.

### Analysis

Several SNA metrics were used to investigate the structural properties of the target networks. For each network, we focused mainly on the number of nodes, density [21], diameter [22], path length [23], clustering coefficient [24], assortativity [25], and degree, closeness and betweenness centrality [26]. Density is determined by dividing the observed number of links in the network by the maximum possible number, which provides a more comprehensive description of the level of connectivity in the network [25]. The greater the density, the more nodes are connected in the network [21]. Diameter is the longest path between two nodes in the network [22]. The average path length of a network is defined as the average distance between all node pairs, including between a node and itself [21]. In other words, a network with a small diameter and a short average distance can be considered "compact", while a network with few nodes and a long distance will have a larger diameter [21]. The clustering coefficient quantifies the number of connected triangles present in a network and helps to reveal the characteristics of individual nodes [24]. A high clustering coefficient means that a person connected to two other people is likely to be connected to others, in a triangle. Assortativity is defined as the degree to which a node in a graph is linked to other nodes with a similar number of connections, for example in a "post/view" relationship [27]. In other words, a high degree of assortativity means that people with many connections are linked to other people with many connections, or conversely, people with few connections are linked to people with few connections. By contrast, low assortativity means that connected individuals are connected to many other, less connected individuals [25]. Reciprocity is defined as the degree of mutual interaction between individuals. It is a measure of the proportion of two-way links in a network. Degree centrality is the number of links a node has. Closeness centrality is higher when the inverse of the sum of the distances from a node to all other nodes in the network (closeness centrality) is biased. Closeness centrality indicates the potential independence of nodes in the flow of an information-sharing network, so if there are information exchange relationships among nodes throughout the network, closeness centrality will be high. Betweenness centrality is a measure of centrality based on the degree to which a node mediates the relationships of other nodes. As betweenness centrality is based on the extent to which nodes mediate relationships between other nodes, a network with few mediators has low betweenness centrality. These measures are widely used in SNA [19, 20]. All measures except the number of nodes, diameter and path length were normalized to a (0,1) scale and expressed as values ranging from 0 to 1. The mean and standard deviation (SD) for each network were calculated. Based on the severity of their disease and the need for care, patients were divided into groups of care levels 1–3 (light care requirement) or 4–5 (heavy care requirement) [3]. For each group, annual comparisons of each score were analyzed using the Steel–Dwass test [28].

## Results

The number of groups (patients) with at least one post in years 2018, 2019, and 2020 was 844, 940, and 1063, respectively. In the SNA, 106/135, 79/89, and 57/57 analyzable networks (i.e., those with at least 10 networks) involving care level 1–3/4–5 were identified, respectively. Table 1 shows the number of networks analyzed along with gender and average age of patients who participated in the networks.

**Table 1:** Number of networks and patient demographic 2018–2020

	2018		2019		2020	
	Care levels 1–3	Care levels 4–5	Care levels 1–3	Care levels 4–5	Care levels 1–3	Care levels 4–5
Number of networks	106	135	79	89	57	57
Female percentage	76 71.7%	86 63.7%	56 70.9%	57 64.0%	40 70.2%	37 65.0%
Male Percentage	30 28.3%	49 36.3%	23 29.1%	32 36.0%	17 29.8%	20 35.0%
Average age (SD)	83.1 (10.6)	82.8 (11.3)	83.1 (10.6)	81.7 (11.5)	83.4 (10.8)	82.4 (10.9)

*SD* Standard deviation

Table 2 shows the number and types of healthcare professionals who participated in each of years 2018, 2019, and 2020.

**Table 2:** Number and types of healthcare professionals participating, 2018–2020

	2018	2019	2020
Total	338	435	319
Physician	6	6	5
Clinic nurse	56	68	66
Home care nurse	85	92	87
Pharmacist	11	11	9
Physical therapist	12	13	13
Occupational therapist	11	15	10
Care manager	68	88	48
Medical social worker	3	2	2
Home care worker	7	14	10
Care worker	35	38	42
Others	44	88	27

The average SNA measurements in care levels 1–3 for 2018, 2019, and 2020 are shown in Table 3. Across 2018, 2019, and 2020, the average diameter tended to increase. In contrast, the assortativity, reciprocity and degree centrality tended to become smaller with each year. Other measures indicated no consistent trends.

**Table 3:** Average SNA measures in care levels 1–3, 2018–2020

	2018	2019	2020
	Nursing care level 1–3	Nursing care level 1–3	Nursing care level 1–3
Average node	18.8 (7.92)	19.8 (9.51)	16.5 (7.53)
Density	0.402 (0.165)	0.381 (0.157)	0.392 (0.155)
Diameter	4.255 (2.24)	4.557 (4.13)	5.000 (3.71)
Path length	1.403 (0.181)	1.441 (0.183)	1.418 (0.161)
Assortativity	-0.250 (0.097)	-0.269 (0.085)	-0.274 (0.080)
Clustering coefficient	0.809 (0.115)	0.761 (0.231)	0.774 (0.161)
Reciprocity	0.406 (0.215)	0.398 (0.231)	0.383 (0.237)
Degree Centrality	0.588 (0.143)	0.589 (0.128)	0.552 (0.147)
Closeness Centrality	0.488 (0.191)	0.509 (0.189)	0.482 (0.157)
Betweenness Centrality	0.162 (0.166)	0.209 (0.235)	0.171 (0.147)

*SD* Standard deviation

The average SNA measurements for care levels 4–5 for 2018, 2019, and 2020 are shown in Table 4.

**Table 4:** Average SNA measures in care levels 4–5, 2018–2020

	2018	2019	2020
	Nursing care level 4–5	Nursing care level 4–5	Nursing care level 4–5
Average node	20.0 (7.91)	21.0 (8.56)	20.4 (9.03)
Density	0.441 (0.133)	0.407 (0.127)	0.381 (0.117)
Diameter	4.037 (1.83)	4.247 (1.40)	4.439 (1.69)
Path length	1.346 (0.164)	1.403 (0.161)	1.414 (0.161)
Assortativity	-0.228 (0.104)	-0.235 (0.094)	-0.260 (0.085)
Clustering coefficient	0.825 (0.096)	0.807 (0.146)	0.778 (0.175)
Reciprocity	0.444 (0.180)	0.443 (0.183)	0.375 (0.169)
Degree Centrality	0.559 (0.105)	0.582 (0.099)	0.585 (0.133)
Closeness Centrality	0.423 (0.143)	0.451 (0.146)	0.472 (0.160)
Betweenness Centrality	0.109 (0.110)	0.140 (0.149)	0.145 (0.145)

*SD* Standard deviation

Across 2018, 2019, and 2020, the average diameter and path lengths, degree centrality, closeness centrality, and betweenness centrality tended to increase. In contrast, the average density, assortativity, clustering coefficient, and reciprocity tended to diminish with each year. Other measures indicated no consistent trends.

Concerning year-to-year differences as assessed by the Steel–Dwass test, there was an overall annual trend toward increasing diameter, but it was not significant. For patients requiring care levels 1–3, assortativity decreased. For those requiring care levels 4–5, reciprocity decreased and betweenness centrality increased significantly. No significant differences were found in the other items.

## Discussion

This study demonstrates how health professionals in Japan have developed the SNS-based information sharing networks in the local community and how the networks vary with the level of care requirement. These annual changes in information sharing networks revealed that networks of patients with a lighter care requirement tended to have reduced assortativity, to which professionals with large and small hub links were connected. By contrast, the networks of patients with heavier care requirements tended to include fixed healthcare professionals who coordinate services in intermediary roles, with reduced interrelationships with other professionals. Network diameters tended to increase regardless of the level of care, but these differences were not statistically significant. The differences and changes in these network indicators are significant for drawing inferences about the potential information sharing mechanisms of healthcare professionals.

Assortativity measures degree of similarity between a node and its neighbors. In a U.S. study, annual changes in assortativity of patient referrals between states were reported as 0.1084 to -0.1217, -0.1104 to -0.1245, 0.0775 to 0.0549, and 0.0800 ~ 0.0569 for in-in, out-out, in–out, and out-in, respectively [29]. The tendency in that study diverges from what we found in the present study, which was that the network tended to be connected through nodes of different degrees. This reflects the fact that about half of the respondents who needed nursing care 1 and 2 were more likely to have increasing care needs than those needing care 4 and 5 [30]. Conceivably, there may be more connections between professionals who were nodes of different levels of care because of changes in care management and services. Networks of patients with heavier care requirements may not show reduced similarity among neighboring nodes compared to those of light-care patients because of the limited number of healthcare professionals who can provide care. In other words, the mechanism appeared to be that the healthcare professionals associated with patients with lighter care requirements are more likely to be connected to a variety of professionals, while those connected to patients with heavier care requirements are more likely to be connected to the same types of professionals.

Reciprocity is useful for understanding two-way relationships because it indicates whether the two nodes contributed equally to a relationship. The reciprocity of the work-related problem-solving network, medication advice-seeking network, and interaction network in an emergency department was reported to be 0.43, 0.26, and 0.24, respectively [31]. Another study reported high reciprocity (0.76) concerning public and private sector networks of midwives [32]. In this study, the reciprocal relationship between health care providers in the network of patients with heavier care requirements was between 0.4 and 0.3, in the mid-range of scores reported in the studies above, but lower over time. This is because patients requiring care levels 4 and 5 were almost always in bed with ADLs, and previous research showed that 70–80% of patients in this group died or experienced deterioration of nursing care level owing to further aging and inactivity after one year [30]. In addition, the network of patients with heavier care requirements tends to include fixed nodes for healthcare professionals that coordinate services in intermediary roles. Intermediary roles in healthcare fields are often practiced as “jobs within jobs” which means that the intermediary operates within the limited rules or regulations decided by the service providers [33]. In these networks, with the intermediary roles fixed, mechanisms to promote smoother interaction may be beneficial.

Density is determined by dividing the actual number of links in a particular network by the total number of nodes, and gives a more comprehensive indication of the level of connectivity in a network [24]. Density is an indicator of shared leadership, [34] reflected in a team's internal network structure [35]. Additionally, it is positively related to team performance and member satisfaction [35]. We found that as the diameter increased, density tended to decrease. This is a result of the indirect addition of new members and the creation of sufficient ties among members to facilitate the flow of information without over-reliance on any one member [36]. Although the density of networks on the internet– such as social networking sites– is often low, team activities have been described as requiring a denser network within a more sparse social structure [37]. In other words, our finding of increasing diameter and decreasing density may be interpreted as the core and periphery network structures beginning to form as new members join the increasingly better-known SNS. A network in which the core and periphery structures are functionally formed has been reported by surgical teams [38]. It is conceivable that similar mechanisms were operating in the year-to-year developments in the SNS-based information sharing network. Patterns of annual changes in SNS-based information sharing networks can be used not only as an analytical tool but also as a means to improve interprofessional collaboration in the context of information technology. Using SNA, future research should aim to clarify the mechanisms underlying development patterns among healthcare professionals, which may contribute to clinical quality improvement.

To further investigate networks with lighter care requirements, it would be important to identify patients whose actual level of care has changed and examine network trends for those patients. Networks involving higher care requirements may be more likely to share information while the intermediary role is more likely to be fixed. This may become apparent in the future research involving comparisons among local communities. These inferred differences in health professionals' information sharing mechanisms by care level requirements provide meaningful evidence for facilitating interprofessional collaboration.

### Strengths and limitations

This study has some limitations. First, the number of nodes was relatively small and may not be representative of Japan, or other local communities, in view of variations in local circumstances including professional healthcare arrangements. Therefore, further validation in other local communities is desirable. In addition, some information sharing might not be reflected in the SNS-based network, as doctors may use tools other than SNS to provide advice to nurses (for example, word of mouth or paper memos). The Japanese healthcare system, in which patients and their families call the visiting nurse first rather than the doctor, may have influenced the findings. However, given the lack of evidence on SNA based information sharing networks among healthcare professionals, this rare example of a longitudinal network survey may contribute to improving the quality of information sharing. Along with analysis of such networks, further research is needed to determine their relationship to patient outcomes, and cost-effectiveness of care-providing systems.

## Conclusion

The findings of this study highlight differences in annual changes in information networks among patients requiring different levels of care. The network of patients with lighter care requirements had more connections with nodes of various sizes, while the those of patients with heavier requirements tended to become characterized by fixed intermediary roles and weaker interrelationships. Clarifying interprofessional collaborative mechanisms underlying development patterns among healthcare professionals can contribute to future clinical quality improvement.

## Data Availability

The data that support the findings of this study are available from the corresponding author [JH], upon reasonable request.

## Abbreviations

SNS: Social network service
SNS: Social network analysis
